# Disseminated gonorrhea presenting as cellulitis of the hand: A case report

**DOI:** 10.1177/2050313X251395983

**Published:** 2025-11-20

**Authors:** Alekhya Gurram, Noah Karnath, Rahul S Nanduri, Khushali Roy, Bernard Karnath

**Affiliations:** 1 John Sealy School of Medicine, University of Texas Medical Branch, Galveston, TX, USA; 2 Department of Internal Medicine, University of Texas Medical Branch, Galveston, TX, USA

**Keywords:** gonorrhea, cellulitis, arthritis, case report

## Abstract

A 35-year-old man presented with left-hand pain, swelling, and erythema. He was initially treated with prednisone, and his symptoms briefly improved but worsened shortly after. Lab tests showed elevated inflammatory markers, and he was referred to rheumatology for suspected rheumatoid arthritis. He later returned to the emergency department with worsening joint pain and painful urination. Imaging revealed muscle swelling without abscess, and he was treated with intravenous antibiotics for presumed cellulitis. Although symptoms improved, cultures later confirmed gonorrhea. He received ceftriaxone and azithromycin at urgent care; then completed intravenous treatment at the hospital. This case illustrates an unusual presentation of disseminated gonorrhea, which typically affects skin and joints, but can mimic cellulitis. Clinicians should consider gonorrhea in the differential diagnosis of unexplained joint pain and swelling, especially when accompanied by urinary symptoms or skin involvement, even in the absence of typical signs of a sexually transmitted infection.

## Background

Gonorrhea, caused by *Neisseria gonorrhoeae*, is a common sexually transmitted infection (STI),^1^ yet disseminated gonococcal infection (DGI) represents a rare complication, occurring in approximately 0.5%–3% of infections.^2^ DGI arises from hematogenous spread and classically presents with a triad of symptoms including pustular skin lesions (dermatitis), tendon inflammation (tenosynovitis), and migratory joint pain and swelling (polyarthritis).^3^ Atypical and severe manifestations such as endocarditis, meningitis, and cellulitis can also occur, although gonococcal cellulitis is extremely rare and difficult to diagnose.^3,4^ Diagnostic delay is common, particularly after prior corticosteroid or empiric antibiotic exposure, which may obscure infectious signs. With rising antimicrobial resistance in *N. gonorrhoeae*, prompt recognition and targeted therapy are essential.^5^ Due to the variable and nonspecific presentations of DGI, healthcare providers should maintain high suspicion for DGI in sexually active patients with unexplained soft tissue or joint infections, even in the absence of genitourinary symptoms.

## Case

A 35-year-old right-handed male construction worker presented to an outside hospital (OSH) with left-hand pain, swelling, and joint stiffness, describing sensations of his “joints locking up.” He was evaluated and prescribed prednisone 50 mg daily, which improved his swelling and resolved erythema. He denied trauma, injury, intravenous drug use in affected hand, and prior joint pain. He also denied systemic symptoms such as fever or chills.

His past medical history was notable for attention-deficit/hyperactivity disorder, anxiety, hypertension, paranoid schizophrenia, intravenous drug use, and opioid use disorder. His medications included amphetamine-dextroamphetamine, atomoxetine, baclofen, buspirone, hydroxyzine pamoate, metoprolol succinate, quetiapine, lisdexamfetamine, and buprenorphine-naloxone. His social history was notable for smoking half a pack of cigarettes per day and being sexually active over the past year (oral and insertive vaginal intercourse) with one female who was recently diagnosed with an ovarian abscess. STI testing history was unknown for the patient and his partner. He also had a history of intravenous drug use, opioid abuse (last use was 8 years ago) and is currently on suboxone. He tested negative for HIV in 2023. He denied any family history of rheumatoid arthritis.

During a visit to another clinic 7 days later, labs were collected. Results showed elevated rheumatoid factor (RF 33 IU/mL) and C-reactive protein (CRP 3.2 mg/dL). Fourteen days after his initial OSH visit, he had a hospital follow-up appointment. Due to concern for early rheumatoid arthritis, he was referred to rheumatology and continued on prednisone.

**Figure 1.**
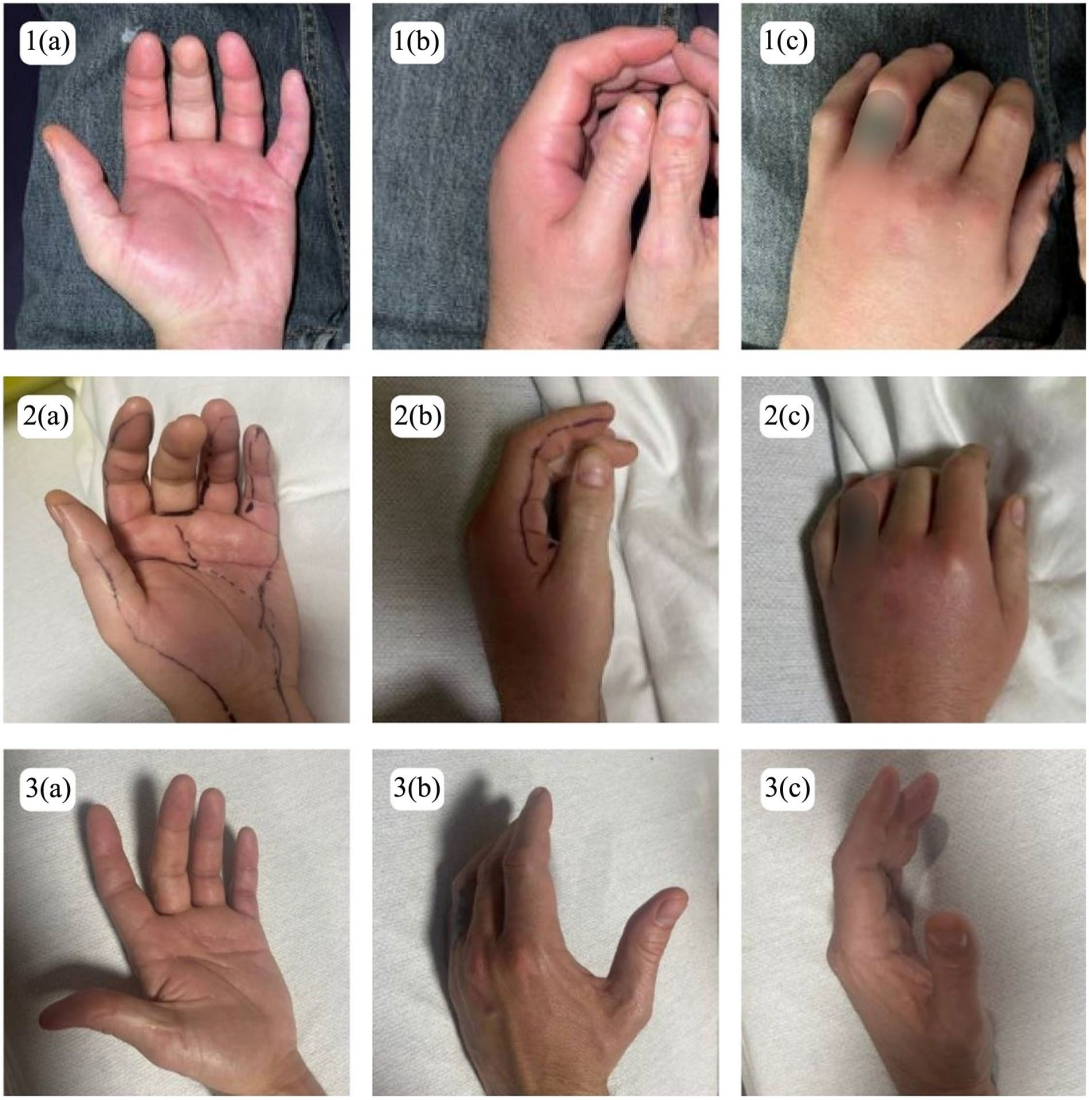
(1a–c): Left-hand cellulitis, 21 days after initial OSH visit. The patient’s tattoo has been digitally obscured to maintain confidentiality. (2a–c): Left-hand cellulitis, 21 days after initial OSH visit. The patient’s tattoo has been digitally obscured to maintain confidentiality. (3a–c): Left-hand cellulitis, 26 days after initial OSH visit. The patient’s tattoo has been digitally obscured to maintain confidentiality.* OSH: outside hospital. *Written informed consent included permission for image publication.

**Figure 2.**
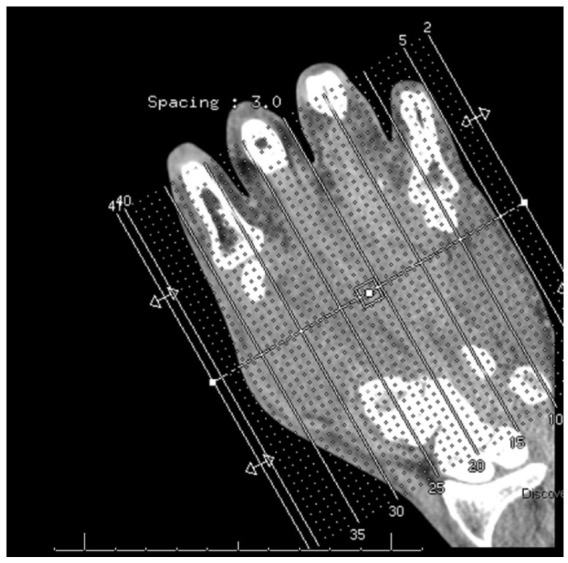
CT left-hand with contrast. No acute osseous abnormality of the left hand. Apparent diffuse prominence of the left thenar muscles, without soft tissue air or drainable fluid collection appreciated. CT: computed tomography.

Twenty-one days after his initial OSH visit, he presented to our emergency department (ED) with worsening left-hand pain and immobility, new joint pain in the right hand and right ankle, and painful urination without urethral discharge. Vital signs on presentation were as follows: blood pressure 148/85 mmHg, heart rate 90 breaths per minute (bpm), respiratory rate 15 bpm, temperature 97.9°F, and oxygen saturation 98% on room air. Physical examination revealed decreased range of motion and swelling of the left hand, without erythema (Figure 1). Computed tomography left hand imaging showed diffuse swelling of the thenar musculature without abscess or fluid collection (Figure 2). Labs revealed leukocytosis, with a white blood count (WBC) of 15.49 × 10^3^/µL; RF was <20 IU/mL, CRP was elevated at 9.2 mg/dL, and antinuclear antibodies (ANA) were positive with a speckled pattern at a titer of 1:80, which is below the threshold typically considered clinically significant. Anti-CCP was not performed, and thus rheumatoid arthritis was not definitively excluded. Given concern for cellulitis or lymphangitis, he was admitted and started on intravenous vancomycin and cefepime. During admission, his swelling improved, but he continued to report joint pains. Blood cultures, urine cultures, and oropharyngeal nucleic acid amplification test (NAAT) were obtained to further investigate infectious etiology of migratory polyarthralgia. HIV and syphilis testing were also obtained.

Three days into his hospital admission following presentation to our ED, which was 24 days after his initial OSH visit, he was discharged in stable condition. Shortly following discharge on the same day, urine cultures returned positive for *N. gonorrhoeae*, while blood cultures and NAAT were negative. HIV and syphilis testing were also negative. He was initially treated at an urgent care facility with a single dose of 1 g intramuscular ceftriaxone and 1 g oral azithromycin. Due to inability to return for daily injections, he was directly admitted to our institution 2 days later (26 days after his initial OSH visit) for continued intravenous therapy. He completed a 6-day course of intravenous ceftriaxone. He developed a headache with no meningeal signs that improved. Additional STI testing, including a throat swab, was negative. Blood cultures were negative 48 h after initiating treatment. At discharge, he declined follow-up but was counseled extensively on STI transmission, treatment adherence, and the importance of partner notification. A timeline of the case is depicted in Table 1.

**Table 1.** Timeline of case.

Day/timepoint	Event/encounter	Details
Day 0	Initial OSH visit	Presented with left-hand pain, swelling, and joint stiffness. Prescribed prednisone 50 mg daily. Denied trauma, injury, or systemic symptoms.
Day 7	Follow-up clinic visit	Labs showed elevated RF (33 IU/mL) and CRP (3.2 mg/dL). Continued on prednisone due to concern for early RA.
Day 14	Hospital follow-up	Referred to rheumatology for suspected RA. Continued on prednisone.
Day 21	ED presentation	Worsening left-hand pain, new right-hand and ankle pain, painful urination. Labs: WBC 15.49 × 10^3^/µL, CRP 9.2, RF <20, ANA 1:80. Imaging showed swelling. Admitted for IV antibiotics (vanc + cefepime).
Day 24	Discharged from hospital	Swelling improved but joint pains persisted. Sent home pending culture results.
Day 24	Culture results	Urine culture positive for *Neisseria gonorrhoeae*; blood and oropharyngeal NAAT negative. Treated initially with 1 g IM ceftriaxone + 1 g azithromycin at urgent care.
Day 26	Hospital readmission for IV therapy	Completed 6-day IV ceftriaxone course. Developed mild headache, resolved. STI testing (throat swab, HIV, syphilis) negative. Discharged after improvement; declined follow-up but received STI counseling.

STI: sexually transmitted infection; OSH: outside hospital; RF: rheumatoid factor; CRP: C-reactive protein; ED: emergency department; WBC: white blood count; ANA: antinuclear antibodies; NAAT: nucleic acid amplification test.

## Discussion

DGI is a rare but serious complication of *N. gonorrhoeae* bacteremia, typically presenting as a triad of migratory polyarthritis, tenosynovitis, and dermatitis.^3^ In this case, DGI presented atypically as unilateral hand swelling and soft tissue inflammation resembling cellulitis. The imitation of bacterial cellulitis complicated early recognition, especially in the absence of genitourinary symptoms.

A notable factor contributing to the diagnostic challenge was the patient’s reported monogamous relationship with a single female partner over the past year, coupled with an unknown personal and partner STI testing history. This might have lowered initial suspicion for a STI and led to delays in pursuing appropriate testing for *N. gonorrhoeae.* STI-related diagnoses should not be excluded solely based on patient-reported sexual history, especially when the partner’s history is incomplete.

Further complicating the diagnosis were early laboratory findings such as elevated RF and CRP, which misleadingly suggested autoimmune pathology such as early rheumatoid arthritis. Treatment with prednisone may have transiently reduced inflammation, masking the underlying infection and facilitating further dissemination of the disease.^6^ The initial clinical improvement on prednisone likely reinforced the erroneous autoimmune diagnosis, delaying appropriate antimicrobial therapy. This highlights the importance of excluding infectious etiologies before initiating corticosteroids in cases of atypical or unexplained soft tissue swelling. In similar scenarios, clinicians should prioritize obtaining baseline inflammatory markers, cultures, and targeted imaging before starting empiric steroids.

Blood cultures can also be negative due to the intermittent nature of gonococcal bacteremia.^7^ In this case, isolation of *N. gonorrhoeae* from urine, despite negative oropharyngeal NAAT results, may reflect sampling after partial antibiotic exposure or localization to the genitourinary tract before hematogenous spread. Therefore, when DGI is suspected, NAAT and culture specimens from multiple mucosal sites, in addition to culture specimens from disseminated sites of infection (skin, blood, cerebrospinal fluid (CSF), and synovial fluid), should be collected and processed to improve diagnostic yield according to the CDC Health Alert Template for DGI.^8^ Early empiric antibiotic therapy with ceftriaxone and azithromycin is recommended in cases of unexplained polyarthritis or soft tissue inflammation in sexually active individuals, as per the 2021 CDC STI Treatment Guidelines for Gonococcal Infections.^9^

Gonococcal cellulitis has been reported in uncommon locations such as the orbit and penis; however, extremity involvement (particularly of the hand) is exceptionally uncommon. Adamson et al. described a 43-year-old female with gonococcal preseptal cellulitis presenting with periorbital pain, swelling, and purulent discharge, in whom urine NAAT confirmed *N. gonorrhoeae*.^10^ She was treated with intravenous ceftriaxone and azithromycin, followed by oral cefixime, with full recovery after a 10-day course.^10^ Lal and Rapose reported a 33-year-old male with penile cellulitis and abscess secondary to *N. gonorrhoeae* infection, who required incision and drainage.^11^ He then completed a 7-day course of ceftriaxone, azithromycin, and oral amoxicillin-clavulanate, also with complete resolution.^11^ Yoshino et al. described the only documented case of a 37-year-old male with multifocal cellulitis of the hand and foot due to disseminated *N. gonorrhoeae* infection, initially treated empirically with piperacillin-tazobactam before culture results prompted a switch to ceftriaxone and adjunctive levofloxacin for concurrent *Chlamydia trachomatis* urethritis, resulting in full recovery after a 14-day course.^12^ In contrast, the present case involved isolated hand cellulitis without multifocal involvement, initially masked by corticosteroid exposure and delayed diagnosis. The patient required readmission for intravenous ceftriaxone after incomplete response to single-dose therapy, completing a 6-day course with full recovery. This case adds to the very limited literature on gonococcal cellulitis and broadens the recognized clinical spectrum of disseminated gonorrhea.

## Conclusion

This case demonstrates that disseminated gonorrhea can present initially as localized hand cellulitis in the absence of genitourinary or systemic symptoms. Clinicians should maintain a high index of suspicion for DGI in sexually active individuals with unexplained soft tissue inflammation and interpret nonspecific inflammatory markers with caution. They should also avoid premature use of corticosteroids without first ruling out infectious causes. In addition to clinical management, this case underscores important public health considerations, including the need for partner notification, comprehensive STI testing, and awareness of emerging antimicrobial resistance in *N. gonorrhoeae.* These steps are essential for preventing reinfection, reducing transmission, and maintaining treatment efficacy.
